# Relevance of Breast Cancer Resistance Protein to Pharmacokinetics of Florfenicol in Chickens: A Perspective from In Vivo and In Vitro Studies

**DOI:** 10.3390/ijms19103165

**Published:** 2018-10-15

**Authors:** Yang Liu, Li Guo, Mire Zloh, Yujuan Zhang, Jinhu Huang, Liping Wang

**Affiliations:** 1 Joint International Research Laboratory of Animal Health and Food Safety, College of Veterinary Medicine, Nanjing Agricultural University, No. 1 Weigang, Nanjing 210095, China; 2016107021@njau.edu.cn (Y.L.); 2014107023@njau.edu.cn (L.G.); 2016807009@njau.edu.cn (Y.Z.); jhuang@njau.edu.cn (J.H.); 2 Faculty of Pharmacy, University Business Academy, Trg Mladenaca 5, 21000 Novi Sad, Serbia; zloh@live.co.uk

**Keywords:** florfenicol, chicken breast cancer resistant protein, substrate

## Abstract

Florfenicol (FFC) is a valuable synthetic fluorinated derivative of thiamphenicol widely used to treat infectious diseases in food animals. The aims of the study were to investigate whether FFC is a substrate for the breast cancer resistance protein (BCRP) and whether the transporter influences oral availability of FFC. In vitro transport assays using MDCK-chAbcg2 cells were conducted to assess chicken BCRP-mediated transport of FFC, while in vivo pharmacokinetic experiments with single or combined BCRP inhibitor gefitinib were employed to study the role of BCRP in oral FFC disposition. According to U.S. Food and Drug Administration (FDA) criteria, FFC was found to be a potential BCRP substrate due to the net efflux ratio being over 2.0 (2.37) in MDCK cells stably transfected with chicken BCRP and the efflux completely reversed by a BCRP inhibitor (Gefitinib). The molecular docking results indicated that florfenicol can form favorable interactions with the binding pocket of homology modeled chicken BCRP. Pharmacokinetic studies of FFC in different aged broilers with different expression levels of BCRP showed that higher BCRP expression would cause a lower Area Under Curve (AUC) and a higher clearance of FFC. In addition, more extensive absorption of florfenicol after the co-administration with gefitinib (a BCRP inhibitor) was observed. The overall results demonstrated that florfenicol is a substrate of the chicken breast cancer resistant protein which in turn affects its pharmacokinetic behavior.

## 1. Introduction

Breast cancer resistance protein (BCRP) is one of the most important ATP-binding cassette (ABC) superfamily transporters for clinically relevant drugs. It is a product of the Abcg2 gene, which encodes 655 amino acids and a 72 kDa protein with six transmembrane (TM) regions and one nucleotide-binding domain (NBD) [1]. With the exception that BCRP was discovered in cancer cells [1,2] and could recognize mainly hydrophilic anticancer agents [3,4], it was also found expressed at pharmacologically important sites such as the apical membranes of enterocytes, hepatocytes, and renal tubular epithelial cells, where they can limit gastrointestinal absorption or mediate direct intestinal, hepatic, or renal excretion of its substrates [5]. Therefore, it may be one of the critical factors limiting the bioavailability of its substrate drugs. Several marketed drugs including sulfasalazine, rosuvastatin, gefitinib, and atorvastatin have been identified as its substrates, and there is increased systemic exposure due to impaired BCRP activity in carriers of the functional polymorphism [6,7]. The herbal-derived compound curcumin is as an inhibitor of BCRP and could increase Cmax and relative bioavailability of sulfasalazine in mice, which demonstrates that BCRP is also an important mediator of drug-drug interactions (DDIs) [8]. Hence, in order to predict the effects of BCRP on drugs pharmacokinetics behaviors and the potential drug-drug interactions, it is necessary to identify if the routinely administered drugs such as antibiotics are substrates and/or inhibitors of BCRP. The U.S. Food and Drug Administration (FDA) has also recommended the assessment of whether a drug candidate is a substrate and/or an inhibitor of BCRP or P-glycoprotein (P-gp) during its development [9].

Florfenicol (FFC) is a fluorinated thiamphenicol derivative and exerts its antibacterial effect by inhibiting protein synthesis at the prokaryotic ribosome [10]. It is frequently used for treating infections caused by a wide range of pathogens including *Escherichia coli*, *Salmonella typhimurium*, *Staphylococcus aureus*, *Streptococcus suis*, *Pasteurella multocida*, and *Actinobacillus pleuropneumoniae* [11,12,13]. Now, it is the major and the only remaining drug of the amphenicols in veterinary-labeled use in China. Florfenicol has been widely used in many species and the pharmacokinetics (PKs) of FFC have been extensively studied in pigs [14], rabbits [15], and broiler chickens [16,17]. However, its reported interaction with a tissue transporter was only limited to P-gp of rabbits [18] and chickens [19], and the function of chicken BCRP on florfenicol metabolism remains to be defined. Given that FFC is an important widely-used antibiotic in veterinary clinics, in this study, we sought to identify whether FFC is a substrate of BCRP whose PK properties are affected by BCRP. 

## 2. Results

### 2.1. Florfenicol is a Substrate of Chicken BCRP Indicated by the Bidirectional Transport Assay in MDCK-chAbcg2 Cells

**Figure 1:**
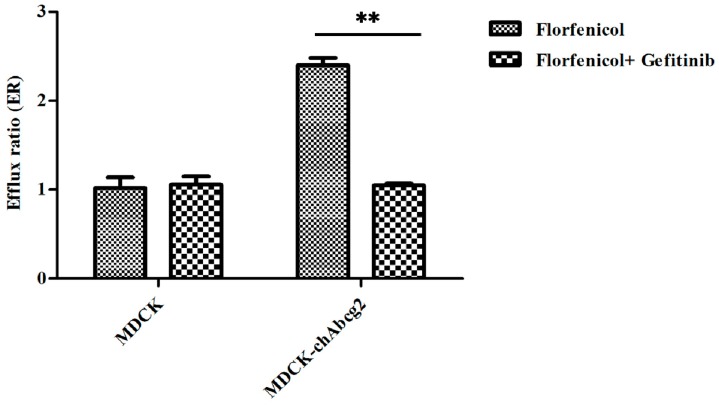
The efflux ratio (ER) of florfenicol across different cell monolayers with or without inhibitor. Data are represented as mean ± SD of three independent experiments. ** *p* < 0.01.

To confirm whether florfenicol is a substrate of chicken BCRP, the bidirectional transport assay of FFC was performed in MDCK (Madin-Darby canine kidney) and MDCK-chAbcg2 cells with or without gefitinib, a BCRP inhibitor. The apparent permeability (*P*_app_), the efflux ratio (ER), and net efflux ratio (NER) of FFC are shown in Table 1. As shown in Figure 1, an efflux ratio (ER) was observed fluctuating from 1.01 to 1.06 when co-administrated with inhibitor (gefitinib) in wild-type MDCK cells and there was no difference (*p* > 0.05). However, in MDCK-chAbcg2 cells, the efflux ratio of FFC dramatically decreased from 2.40 to 1.15 by gefitinib (*p* < 0.001), accordingly, NER significantly dropped from 2.37 to 1.09 (*p* < 0.01). NER was more than 2 (2.37), meanwhile, BCRP inhibitor could reverse the BCRP-medicated transport, which indicated that FFC was the substrate of chicken BCRP. 

### 2.2. Florfenicol Might Favorably Bind with BCRP Analyzed by Molecular Docking Modelling

**Figure 2:**
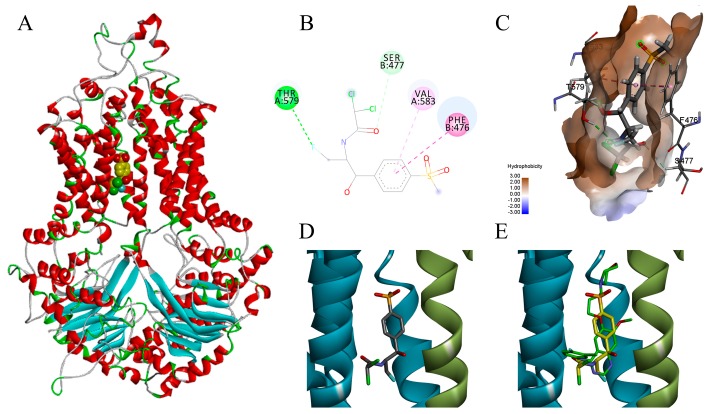
Molecular docking of florfenicol into the cBCRP target obtained using AutoDock Vina. (**A**) Homology model of the BCRP (ribbon representation colored according to secondary structure) and florfenicol (CPK(Corey–Pauling–Koltun) representation). (**B**) ligand interaction diagram of the florfenicol docking pose (dark green—conventional hydrogen bond; light green—carbon hydrogen bond; dark pink—pi-pi stacked interaction; light pink—pi-alkyl interaction; yellow—pi-sulfur interaction). (**C**) Florfenicol in the active sites delineated by the hydrophobic surface and surrounding residues which are labelled and represented as thin grey lines(dashed lines represent the same interactions as in B). Hydrogen atoms are not shown for clarity. (**D**) Florfenicol (thick grey lines) in the ligand binding domain of the cBCRP (ribbon representation: chain A colored in green and B in cyan). (**E**) Florfenicol (thick yellow lines) overlaid onto gefinitib (thick green lines) in the ligand binding domain of the cBCRP (ribbon representation: chain A colored in green and B in cyan).

The homology model of chicken BCRP (Figure 2A) was compared to a homology model of human BCRP reported previously [20] as the experimental structure of this transporter is not currently available. Both structures were similar in terms of secondary structure and tertiary fold with an overall RMSD of 2.56 Å between two structures. This has suggested that chicken BCRP (cBCRP) homology model should be suitable to be used to evaluate affinity of florfenicol towards the binding site of its ligand binding domain. Furthermore, docking was carried out for a number of known BCRP substrates (ampicillin, ciprofloxacin, clindamycin, enrofloxacin, imatinib, irinotecan, lapatinib, methotrexate, mitoxantrone, rosuvastatin, sulfasalazine and topotecan) and inhibitors (elacridar, gefitinib, and eltrombopag) into the efflux pump site. The similar docking scores obtained for substrates except for the irinotecan (Table 2) along with their similar space occupied inside the binding pocket (data not shown) confirmed the model suitability for exploring the binding modes of cBCRP substrates. Furthermore, the binding poses of known BCRP inhibitors show that they generally bind more favorably and occupy a larger space of the binding pocket (Figure 2B–D). In particular, the docking score best pose obtained for florfenicol was −8.3 kcal/mol, while the docking score for gefitinib of −9.6 indicated more favorable binding, suggesting a possible competitive inhibition of cBCRP (Figure 2E). 

### 2.3. Age-Dependent mRNA Expression of BCRP in Kidney and Jejunum in Broilers

**Figure 3:**
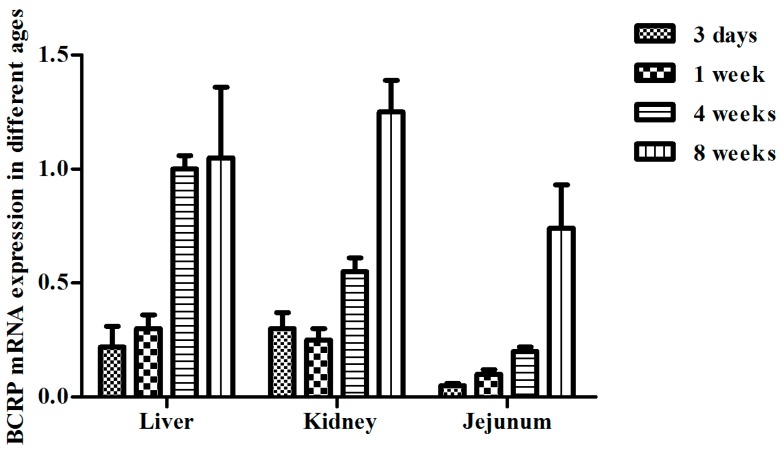
Comparison of the BCRP mRNA levels in different ages. Data are represented as mean ± SD (*n* = 5).

**Figure 4:**
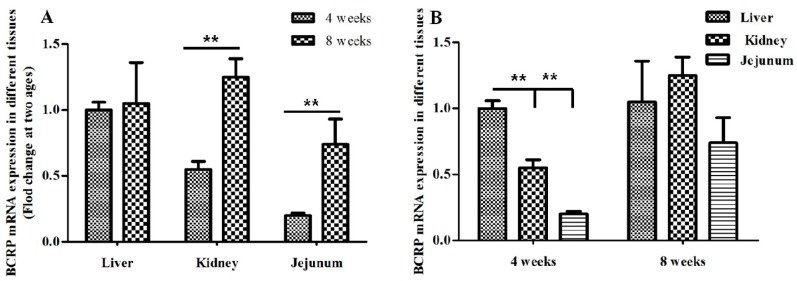
Comparison of the BCRP mRNA level in different tissues in broilers at different ages. (**A**) Comparison of the BCRP mRNA levels in same tissues in broilers at different ages. (**B**) Comparison of the BCRP mRNA levels in different tissues in 4- and 8-week old broilers. Data are represented as mean ± SD (*n* = 5). ** *p* < 0.01.

With regard to BCRP expression in broilers during ontogeny, we measured the mRNA expression of Abcg2 in broilers at ages from day 1 to 8 weeks, which corresponds to the stages in broiler husbandry. As shown in Figure 3, the expression level of Abcg2 mRNA varied with age, however, the expression level of Abcg2 mRNA was very low in the liver or small intestines from those very young birds. Therefore, we focused on studies of broilers aged from 4 to 8 weeks. According to the real-time RT-PCR results, the patterns of mRNA expression in the kidney and jejunum were age-dependent. As shown in Figure 4A, the broilers at 8 weeks of age expressed significantly higher Abcg2 mRNA levels in the kidney and jejunum than at the age of 4 weeks (*p* < 0.01). However, no significant age-related difference was found in the liver (*p* > 0.05), although little fluctuation was observed within each age group. At the same time, there were difference among tissues in same age, as shown in Figure 4B, the highest expression is the liver, followed by the kidney, and the lowest is the small intestine at the age of 4 weeks. Though there were no significant variations, a fluctuation was also observed among tissues in same age with highest mRNA level in the kidney at the age of 8 weeks. 

### 2.4. Pharmacokinetic Behavior of Florfenicol Is Different in Broilers at Different Ages

**Figure 5:**
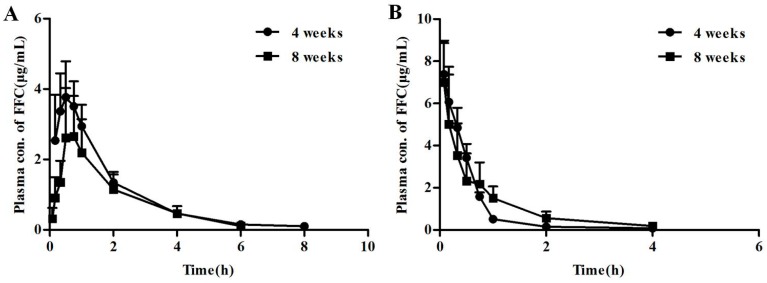
The concentration-time profiles of florfenicol. (**A**) The plasma concentration-time profiles of orally administered florfenicol (20 mg/kg b.w.) in the 4- and 8-week old broilers. (**B**) The plasma concentration-time profiles of intravenous administered florfenicol (10 mg/kg b.w.) in the 4- and 8-week old broilers. All data represent mean ± SD (*n* = 5).

It may be viable that the different expression levels of BCRP in 4- and 8-week old broilers affect the pharmacokinectics of FFC in broilers of different age. To test it, the broilers at 4- and 8-weeks of age were selected to conduct the pharmacokinetic experiment, based on the different levels of Abcg2 mRNA expression in the kidney and intestines of the birds. The plasma concentration-time profiles of florfenicol after a single oral (20 mg/kg) or intravenous (10 mg/kg) administration of florfenicol in broilers at different ages are illustrated in Figure 5, and the relevant pharmacokinetic parameters are shown in Table 3 and Table 4. The 8 week old broilers, when orally administered florfenicol (Figure 5A), exhibited a significantly higher *V*d (5.64 vs. 3.42 (mg/kg)/(µg/mL), *p* < 0.01), Cl (4.43 vs. 2.43 mg/kg/h/(μg/mL), *p* < 0.05) as well as a lower AUC_0~12h_ (4.91 vs. 8.58 (µg/mL)·h, *p* < 0.01) and *T_1/2β_* (0.94 vs. 3.27 h, *p* < 0.05), compared with those in 4 week-old broilers. In addition, the bioavailability of florfenicol in 8-week old broilers was decreased by 31.3% compared with that in 4-week old birds. However, when florfenicol was *i.v.* administered, there were no significant differences of the all parameters between the two groups of broilers (Table 4). These results suggest that the higher expression of BCRP possibly affects the pharmacokinetics of florfenicol, leading to decreased bioavailability of florfenicol in the 8-week old broilers.

### 2.5. BCRP Inhibitor Gefitinib Affected the Pharmacokinetics of Florfenicol Orally Administrated in Broilers

**Figure 6:**
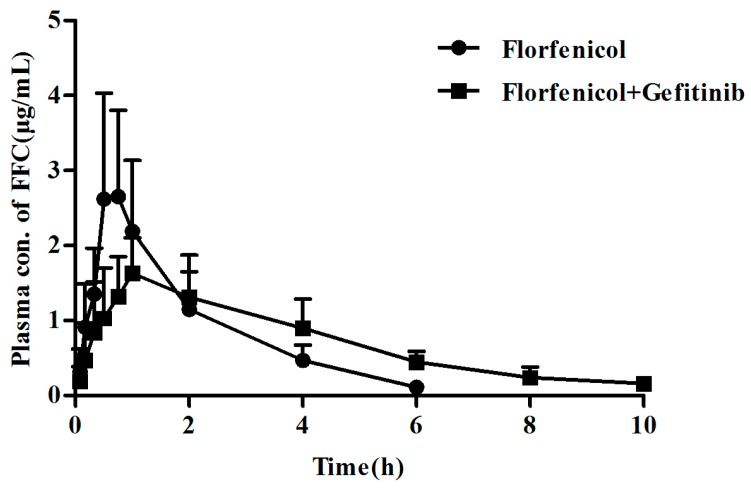
The plasma concentration-time profiles of orally administered florfenicol (20 mg/kg b.w.) in the presence and absence of gefitinib (100 mg/kg). Data represent mean ± SD (*n* = 5).

To substantiate the effect of BCRP expression level on the pharmacokinetics of florfenicol in 8-week old broilers, gefitinib, an inhibitor of BCRP, was employed. The mean plasma concentration-time profiles of single-oral florfenicol (20 mg/kg) in the presence or absence of oral gefitinib (100 mg/kg, single dose) are shown in Figure 6. The relevant pharmacokinetic parameters are listed in Table 3. The combination of florfenicol and gefitinib exhibited a significantly higher AUC_0~12h_ (8.01 vs. 4.91 (µg/mL)·h, *p* < 0.05), as well as a lower Cl (2.57 vs. 4.43 mg/kg/h/(μg/mL), *p* < 0.05), accordingly, increasing the bioavailability to 78.4%, compared to florfenicol alone. The results further suggested that the level of BCRP impacts the pharmacokinetics of orally administered florfenicol in broilers. 

## 3. Discussion

Breast cancer resistance protein is an important transporter expressed in normal tissues of animals and may play a direct clinical role in the disposition of drugs and in explaining certain clinical drug-drug interactions [8,21,22]. To the best of our knowledge, this study is the first to profile the age-dependent patterns of BCRP mRNA expression in the liver, kidney, and jejunum of the chickens and the homology model of the cBCRP was also firstly generated and validated by our group. Of greater importance, our findings are among the first to strongly suggest that BCRP had an effect on the pharmacokinetic behavior of florfenicol, a substrate of BCRP screened by bidirectional transport in MDCK cells stably transfected with chicken Abcg2 and molecular docking.

Understanding the age-associated alterations of BCRP expression in absorptive and excretive organs is important for the evaluation of therapeutic efficacy in juvenile and adult animals. Limited studies have examined the ontogenic expression of BCRP in rats and humans, but discrepant observations were reported [23,24,25], for example, BCRP expression levels were low in fetal kidneys, increased gradually following birth, and markedly increased on maturation and adulthood in rats [23], which is similar with our results in chicken kidney. The levels of BCRP did not differ regionally in adult human livers, but decline in the elderly [24]; however, no obvious age-related changes have been found in the liver of chickens. Therefore, knowledge of the age-related variation of BCRP in chicken cannot simply be interspecies extrapolated and needs to be determined for chickens. The factors responsible for the age-dependent changes in BCRP expression are still unclear. However, Demeule et al. [26] and Iqbal et al. [27] have independently demonstrated that the expression of P-gp is upregulated by steroids and other hormones whose activity may change during aging. We speculated it is similar for BCRP.

According to FDA guidance [9], if a drug with a net efflux ratio that is over 2.0 and its efflux is significantly inhibited by one or more BCRP specific inhibitors, the drug can be considered as a potential substrate of an efflux pump. In this study, FFC net efflux ratios (NERs) were detected as 2.37 through wild-type MDCK cell line and MDCK-chAbcg2 cell line which was previously generated in our lab by using lentiviral vector system to transfected chicken *Abcg2* gene encoding BCRP [28]. The NER value of FFC significantly decreased from 2.37 to 1.09 by BCRP inhibitor gefitinib which is similar to the effect of another BCRP inhibitor Ko143 [28], suggesting that FFC is involved in BCRP-mediated efflux. The molecular docking study shows that florfenicol can bind into the binding pocket of chicken BCRP (Figure 2B,C), forming the favorable interactions with residues of both chains (chain A: THR579 and VAL583; chain B: PHE476 and SER477). The aromatic ring of florfenicol forms π-π and π-σ interactions with neighboring residues. The docking score for florfenicol is similar to the values of those obtained for the known BCRP substrates, while being lower than docking score of inhibitors (Table 2). The inhibitors generally occupy larger space inside the binding site and form favorable interactions with more residues than substrates. Particularly in the case of the drug (florfenicol), efflux pump inhibitor (gefitinib) pair, the inhibitor interacts with the same residues as the florfenicol, and forms two additional hydrogen bonds with tertiary amine of the morpholine ring and nitrogen in the quinazoline ring. It is highly likely that gefitinib inhibits the efflux of florfenicol by competitive binding, although other mechanisms cannot be neglected. Therefore, the results of the docking studies also suggest that florfenicol could be a substrate for the BCRP mediated efflux. 

It has to be taken into consideration that gefitinib can also be a BCRP substrate at lower concentrations [29], thus the current experiments were conducted using high concentrations of gefitinib to ensure its inhibitory activity against BCRP. At this concentration, there is a possibility that there are off-target interactions that may contribute to observed results. The attempts to conduct in vivo experiments using an additional BCRP inhibitor, Ko143, were unsuccessful due to its observed toxicity in chicken, and more appropriate additional inhibitor should be considered in the future studies to confirm these effects beyond gefitinib.

It might be due to different pharmaceutical formulations, or different ages or breeds of birds used in the studies, the main pharmacokinetic parameters of oral FFC in the broilers (4- and 8-week old) of the current study were not completely in accordance with the values in other studies [16,17,30]. However, significant different pharmacokinetic behavior of orally administrated FFC in 4- and 8-week old broilers with various levels of BCRP were observed in this study. It is obvious that AUC_0__~12__h_ and bioavailability was higher in younger birds (4-week) than older ones and the body clearance rate is higher in older birds than that in younger ones. But there were no significant differences in intravenously administrated in 4- and 8-week old broilers, which implied that the increase of bioavailability is due to a higher FFC absorption from small intestine in 4-week old broilers. Published papers have suggested that FFC can be absorbed from the small intestine and is primarily eliminated by renal excretion as an unchanged compound (42%) and its metabolite florfenicol amine (25%) [31,32]. Considering that FFC is a substrate of BCRP, the modulation of BCRP expression level and activity may cause significant changes in the pharmacokinetic profiles of FFC. Therefore, it can be concluded that the potential role of BCRP involved in FFC disposition in broilers and it is further supported by the finding that gefitinib, an inhibitor of BCRP, was able to increase the bioavailability of FFC from 48.04% to 78.38%. Cmax did not obviously change, however, Cl has significantly decreased by gefitinib, which implied that the increase of bioavailability is due to the decreased elimination of FFC by the kidney through reversing the efflux function of kidney BCRP by gefitinib. 

In conclusion, both in vitro and in vivo results demonstrate that florfenicol is a substrate for BCRP. If we consider the increasing threat of antimicrobial resistance and the possible role of veterinary use of antimicrobials in its emergence, then it seems obvious that much greater attention should be paid to the development of more precise and safer dosage regimen based on age-associated BCRP levels, to promote the concept of prudent use of antimicrobials such as florfenicol in poultry.

## 4. Materials and Methods

### 4.1. Reagents

Florfenicol (FFC) (pure active pharmaceutical ingredient) was kindly provided by China Institute of Veterinary Drug Control (Beijing, China). Gefitinib (pure active pharmaceutical ingredient), as a BCRP inhibitor, was purchased from MedChemExpress (Monmouth Junction, NJ, USA). Reverse transcription kit, SYBR green was purchased from Takala (Tokyo, Japan). All other chemicals were of analytical grade and obtained from standard suppliers unless mentioned otherwise.

### 4.2. Cell Lines

Wild-type MDCK cells were purchased from Shanghai Institute of Cell Biology and maintained in Dulbecco’s modified Eagle’s medium (DMEM) supplemented with 10% fetal bovine serum, 1% glutamine, and 1% penicillin at 37 °C and 5% CO_2_. MDCK-chAbcg2 cell line stably expressing chicken BCRP was established in our laboratory [28]. The cell lines were cultured as the above and supplemented with 1.0 μg/mL puromycin.

### 4.3. Animals

Arbor Acres (AA) broilers (1-day old from the same brood) were purchased from a local commercial poultry farm (Nanjing, China). The broilers were given basal diet (without antibiotics and coccidiostats) and water, ad libitum, and managed under recommended humidity and temperature for 4~8 weeks. All procedures regarding handling of broilers were approved (code 2018-0035, 15/02/2018) and followed by the Animal Care and Use Committee of College of Veterinary Medicine, Nanjing Agricultural University (SYXK-Su 2017-0007, Nanjing, China). At the age of 4 and 8 weeks, respectively, some chickens were used to evaluate mRNA expression and while the rest of the chickens were used to conduct pharmacokinetics experiments. 

### 4.4. Bidirectional Transport Experiments in MDCK-chAbcg2 Cells

Wild-type MDCK cells and MDCK-chAbcg2 cells were seeded into Transwell^®^ inserts (Catalogue number 3460, Corning Coster Corp. Acton, MA, USA) in 12-well plates at a density of 1 × 10^5^ cells/insert and cultured for 5 days. The cell layer integrity was tested before transport by measuring transepithelial electrical resistance (TEER) across the apical (AP) and the basolateral (BL) sides of the cell line using a Millicell-ERS epithelial voltometer (Millipore, Burlington, MA, USA). The bidirectional transport experiments were conducted with the transport buffers containing either FFC (50 μM) or the mixture of FFC and gefitinib instead of the blank medium or the medium contain gefitinib (100 μM). After the cells were incubated with transport buffers for 4 h, the samples collected from the receiving sides were submitted for HPLC analysis. The TEER values of each monolayer were checked again at the end of the transport studies to ensure the integrities of cell monolayers during the whole transport analysis. According to FDA guidance [9] a drug with a net efflux ratio that is over 2.0 will be considered as a potential substrate of an efflux pump. The apparent permeability coefficients (*P*_app_) across cell monolayer were calculated as follows: *P*_app_ = (*dQ*/*dt*)/(*A* × *C*_0_), where *A* is the area of filter membrane, *C*_0_ is the initial concentration of the tested drug, *dQ* is the amount of transported drug, and *dt* is time elapsed. The efflux ratio (ER) was calculated from (*P*_app_ B→A)/(*P*_app_ A→B) and the net efflux ratio (NER) was calculated from *ER*_MDCK-chAbcg2_/*ER*_MDCK_.

Each group has 5 cell monolayers, and the experiment was repeated three times.

### 4.5. Molecular Modelling

Structures of florfenicol and known BCRP substrates (ampicillin, ciprofloxacin, clindamycin, enrofloxacin, imatinib, irinotecan, lapatinib, methotrexate, mitoxantrone, rosuvastatin, sulfasalazine, and topotecan) as well as inhibitors (elacridar, gefitinib, eltrombopag) were collated as canonical SMILES (Simplified Molecular Input Line Entry System) string from PubChem database [33] and their starting structures were generated from these SMILES strings using Avogadro molecular modelling software version 1.2.0 [34]. Furthermore, protonation states of ionizable groups of molecules were set to reflect their dominant charge states at pH 7.4 using Avogadro. The lowest energy conformation for each molecule was calculated using the same software and MMFF94 force field and saved in a mol2 file format. 

Homology model of the cBCRP was generated using procedure described previously [35] with some modifications. Then, model availability was validated by comparing docking scores of known BCRP substrates and inhibitors into the efflux pump site. Briefly, the GenPept database was used to retrieve its sequences with the accession number NP_001315419.1, which was submitted to SWISSPROT homology modelling server [36,37] to obtain its three-dimensional structure. Only one model was generated using the known structure of the human multidrug transporter ABCG2 (PDB entry 6FFC) [38] as a template. The generated homology model was superimposed onto the 6FFC structure using Maestro Graphic User Interface v2018-1. The two copies of the ligand BWQ were copied into the binding site of the homology model and the steric clashes were removed manually. Furthermore, the complex was prepared for molecular dynamics simulation using Protein Preparation wizard by adding all hydrogen atoms and setting the protonation states of all ionizable groups in the protein for pH 7 [39]. The molecular simulations were performed with Desmond [40] and OPLS2005 force field. The efflux pump model was embedded into POPC (1-palmitoyl-2-oleoyl-sn-glycero-3-phosphocholine) membrane model and was fully solvated using an explicit solvent (Simple point charge (SPC) water model) with the box size 10 Å larger than the size of a protein in all directions using System Builder. The system was automatically neutralized by addition of an adequate number of relevant ions and minimized until the norm of the energy gradient was <0.1 kcal/mol. Furthermore, the whole system was simulated for 1 ns at 300 K under constant pressure and temperature (NPT) conditions. The structure of the cBCRP extracted from the final frame was used for further molecular docking.

AutoDock Vina software (version 1.1.2) [41] implemented in VegaZZ scripting environment [42] was used for docking of florfenicol into the active sites of cBCRP. The binding sites, with the sizes of 24 Å × 24 Å × 24 Å, were positioned in the geometrical centers of a bound ligand that was removed from the structure prior to docking. Exhaustiveness was set to 20, and the top nine favorable binding modes were calculated for each molecule. All images were generated using Biovia Discovery Studio 2016. 

### 4.6. BCRP mRNA Expression in Tissues of Broilers at Different Ages by Real Time RT-PCR

The mRNA expression level in liver, kidney and jejunum in broilers at the age of 4- and 8-week old broilers (5 birds/group) were evaluated by real time RT-PCR, respectively. Total RNA was isolated from individual tissues of all birds using Trizol Reagent (Takara, Tokyo, Japan) according to the manufacturer’s instructions. The total RNA concentration was then quantified by Nanodrop photometer (ND-1000 Spectrophotometer, Rockland, DE, USA). Ratios of absorption (260/280 nm) of all preparations were between 1.8 and 2.0. And then 100 ng RNA were treated with gDNA Eraser (Takara, Tokyo, Japan) for 2 min at 42 °C to ensure that all total RNA was free of genomic DNA contamination. Single-stranded cDNAs were synthesized using the reverse transcription kit and real-time PCR was performed, as instructions described. Primers specific for BCRP and β-actin were designed as described in Table 5 and commercially synthesized by Shanghai Generay Biotech Co. Ltd. (Shanghai, China). Chicken β-actin was chosen as a housekeeping gene for normalization. The 2^−ΔΔCt^ method [43] was used to analyze the real-time RT-PCR data.

### 4.7. Pharmacokinetic Studies of FFC in Broilers

#### 4.7.1. Experiment Design and Blood Collection

When the chickens reached the age of 4 weeks and 8 weeks old, the pharmacokinetics test were conducted. Ten 4-week and fifteen 8-week old AA broilers were used in this study and divided into five groups (5 birds in each group) as follows: Group I, 4-week old birds given FFC orally with a single dose of 20 mg/kg through crop tube gavage; group II, 8-week old birds given FFC orally with a single dose of 20 mg/kg through crop tube gavage; group III, 4-week old birds given FFC intravenously with a single dose of 10 mg/kg through the left brachialis vein; group IV, 8-week old birds given FFC intravenously with a single dose of 10 mg/kg through the left brachialis vein; group V, 8-week old birds orally given FFC (20 mg/kg) plus gefitinib (100 mg/kg, an inhibitor of BCRP) through crop tube gavage. Groups I, II, III, and IV were used to study the influence of age-dependent BCRP expression on FFC pharmacokinetics. The data from group II and group V were used to further analyze the effects of BCRP on FFC pharmacokinetics. Blood samples were taken from the wing vein of each chickens and collected into heparin-coated tubes at 0.083, 0.167, 0.33, 0.5, 0.75, 1, 2, 4, 6, 8, 10, and 12 h after administration of FFC. Plasma was rapidly harvested by centrifugation at 5000 g for 5 min and stored at −80 °C until assayed.

#### 4.7.2. Determination of FFC Concentration in Broilers Serum by HPLC Method

The extraction procedures and HPLC method were modified from the previous method [16]. All plasma samples were analyzed on Thermo fisher U3000 high-performance liquid chromatography (HPLC) system. Frozen plasma (0.2 mL) was thawed at 4 °C and the plasma sample was extracted twice with 1 and 0.5 mL acetonitrile, respectively. The acetonitrile supernatants were combined, evaporated under nitrogen at 40 °C, and dissolved in 0.2 mL of mobile phase, from which 20 μL was injected into HPLC system. The mobile phase consisted of a mixture of acetonitrile-water at a ratio of 20:80 (*v*/*v*). The detection conditions as the follows: wavelength at 224 nm, the column temperature at 35 °C and the flow rate at 1 mL/min. The calibration samples were prepared with eight different FFC concentrations using blank plasma. A linear relationship existed in the calibration curve from 0.1 to 10 μg/mL, which consistently yielded a correlation coefficient >0.99. The average of inter- and intra-day precision (*RSD*) was <11%. At concentrations of 0.1, 1, and 10 μg/mL, the recovery range of FFC was from 100% to 110%. The limit of detection was 0.1 μg/mL. 

#### 4.7.3. Pharmacokinetic Data Analysis

Pharmacokinetic calculations were performed on each individual set of data using 3p97 practical pharmacokinetic software (Version 97, Chinese Pharmacologic Association, Beijing, China). Least-squares fits were determined for both biexponential (two-compartment model) and single exponential (one-compartment model) equations. The number of exponential terms required to describe the plasma concentration-time data for each boiler chickens was determined by application of Akaike’s Information Criterion (AIC Method). The area under the concentration-time curve (AUC) was calculated according to the linear trapezoidal method. The systemic bioavailability (*F* %) of florfenicol after oral administration was determined as follows: *F* = *AUC*_0−_*_t_* *^p.o.^*· Dose *^i.v.^*/*AUC*_0__-*t*_ *^i.v.^* ·Dose *^p.o.^ ×* 100%.

### 4.8. Statistical Analysis

Statistical analysis was performed using SPSS software (version 20.0, SPSS Inc., Chicago, IL, USA) under Student’s *t*-test. *p* < 0.05 was considered as significant difference, and *p* < 0.01 was considered as extremely significant difference. Data were shown as means ± SD.

## Figures and Tables

**Table 1:** Permeability, efflux ratio (ER), and net efflux ratio (NER) of florfenicol (FFC) across different cell monolayers.

Cell Lines	Inhibitor (Gefitinib)	*Papp* (×10^−6^ cm/s)		Efflux Ratio (ER)	*p*-Values	Net Efflux Ratio (NER)	*p*-Values
		AP→BL	BL→AP				
MDCK	−	0.40 ± 0.04	0.40 ± 0.01	1.02 ± 0.12	0.656	-	-
	+	0.37 ± 0.03	0.39 ± 0.01	1.06 ± 0.09		-	
MDCK-chAbcg2	−	0.28 ± 0.01	0.68 ± 0.04	2.40 ± 0.08	0.000	2.37 ± 0.28	0.002
	+	0.37 ± 0.02	0.43 ± 0.01	1.15 ± 0.02 **		1.09 ± 0.09 **	

Mean ± SD, *n* = 3, ** *p* < 0.01, compared between gefitinib treatment and non-treatment.

**Table 2:** Docking scores of breast cancer resistance protein (BCRP) substrates and inhibitors into the efflux pump site.

Molecule	Docking Score (kcal/mol)	Molecule	Docking Score (kcal/mol)
Substrates			
florfenicol	−8.3	ciprofloxacin	−9.9
irinotecan	−12.8	enrofloxacin	−9.4
topotecan	−11.0	ampicillin	−8.7
lapatinib	−11.0	rosuvastatin	−8.6
imatinib	−10.9	mitoxantrone	−8.3
sulfasalazine	−10.7	clindamycin	−8.0
methotrexate	−10.1		
Inhibitors			
eltrombopag	−13.0	elacridar	−12.2
gefitinib	−9.6		

Docking was carried out using Autodock Vina and VegaZZ as a graphical user interface.

**Table 3:** Parameters of florfenicol administrated orally (20 mg/kg) in the 4- and 8-week old broilers (in the presence and absence of gefitinib (100 mg/kg)), respectively.

Parameters	4-Week Old Broilers	8-Week Old Broilers	*p* Values	8-Week Old Broilers Administered Gefitinib	*p*-Values
*C*_max_ (μg/mL)	3.87 ± 0.96	2.93 ± 1.07	0.159	1.73 ± 0.48	0.079
*T*_max_ (h)	0.63 ± 0.21	0.62 ± 0.19	0.943	2.00 ± 1.41	0.064
*T_1/2β_* (h)	3.27 ± 1.20	0.94 ± 0.17 *	0.012	3.50 ± 3.35	0.224
*V*_d_ (L/kg)	3.42 ± 1.18	5.64 ± 0.60 **	0.005	7.39 ± 2.98	0.327
*Cl_B_* (L/h/kg)	2.43 ± 0.53	4.43 ± 1.46 *	0.034	2.57 ± 0.52 #	0.048
*AUC*_0~12h_ (mg·h/L)	8.58 ± 1.88	4.91 ± 1.56 **	0.007	8.01 ± 1.65 #	0.023
F (%)	79.30	48.04	-	78.38	-

Mean ± SD, *n* = 5, * *p* < 0.05, ** *p* < 0.01, compared between 4 and 8-week broilers; # *p* < 0.05, compared between gefitinib treatment and non-treatment. *C*_max_: maximal plasma concentration; *T*_max_: time to obtain *C*_max_; *V*_d_: apparent distribution volume; *Cl_B_*: apparent clearance; *T_1/2β_*: elimination half-life; *AUC*_0~12h_: area under the plasma concentration–time curves; *F*: absolute bioavailability.

**Table 4:** Parameters of florfenicol administered intravenously (10 mg/kg) in the 4- and 8-week old broilers.

Parameters	4-Week Old Broilers	8-Week Old Broilers	*p*-Values
*T_1/2β_* (h)	1.0 ± 0.85	0.79 ± 0.18	0.598
*V*_d_ (L/kg)	1.51 ± 0.62	1.18 ± 0.29	0.310
*Cl_B_* (L/h/kg)	1.65 ± 0.54	2.14 ± 0.67	0.239
*AUC*_0~12h_ (mg·h/L)	5.41 ± 0.31	5.11 ± 1.89	0.741

**Table 5:** Nucleotide sequences of the primers used for real-time quantitative PCR analysis.

Gene	Forward (5′–3′)	Reverse (5′–3′)	Length/bp	Tm (°C)
*β*-actin	ATGTGGATCAGCA AGCAGGAGTA	TTTATGCGCATTT ATGGGTTTTGT	300	54.1
Abcg2	CCTACTTCCTGGCC TTGATGT	TCGGCCTGCTATA GCTTGAAATC	180	56

## References

[1] Mao Q., Unadkat J.D. (2014). Role of the Breast Cancer Resistance Protein (BCRP/ABCG2) in Drug Transport—An Update. AAPS J..

[2] Doyle L.A., Yang W., Abruzzo L.V., Krogmann T., Gao Y., Rishi A.K., Ross D.D. (1998). A multidrug resistance transporter from human MCF-7 breast cancer cells. Proc. Natl. Acad. Sci. USA.

[3] Vlaming M.L., Lagas J.S., Schinkel A.H. (2009). Physiological and pharmacological roles of ABCG2 (BCRP): Recent findings in Abcg2 knockout mice. Adv. Drug Deliv. Rev..

[4] Doyle L., Ross D.D. (2003). Multidrug resistance mediated by the breast cancer resistance protein BCRP (ABCG2). Oncogene.

[5] Maliepaard M., Scheffer G.L., Faneyte I.F., van Gastelen M.A., Pijnenborg A.C., Schinkel A.H., van De Vijver M.J., Scheper R.J., Schellens J.H. (2001). Subcellular localization and distribution of the breast cancer resistance protein transporter in normal human tissues. Cancer Res..

[6] Giacomini K.M., Balimane P.V., Cho S.K., Eadon M., Edeki T., Hillgren K.M., Huang S.M., Sugiyama Y., Weitz D., Wen Y. (2013). International Transporter Consortium commentary on clinically important transporter polymorphisms. Clin. Pharmacol. Ther..

[7] Cusatis G., Gregorc V., Li J., Spreafico A., Ingersoll R.G., Verweij J., Ludovini V., Villa E., Hidalgo M., Sparreboom A. (2006). Pharmacogenetics of ABCG2 and adverse reactions to gefitinib. J. Natl. Cancer Inst..

[8] Kusuhara H., Furuie H., Inano A., Sunagawa A., Yamada S., Wu C., Fukizawa S., Morimoto N., Ieiri I., Morishita M. (2012). Pharmacokinetic interaction study of sulphasalazine in healthy subjects and the impact of curcumin as an in vivo inhibitor of BCRP. Br. J. Pharmacol..

[9] US Food and Drug Administration (2012). Guidance for Industry—Drug Interaction Studies: Study Design, Data Analysis, Implications for Dosing, And Labeling Recommendation.

[10] Sidhu P., Rassouli A., Illambas J., Potter T., Pelligand L., Rycroft A., Lees P. (2014). Pharmacokinetic-pharmacodynamic integration and modelling of florfenicol in calves. J. Vet. Pharmacol. Ther..

[11] Shupeng H., Tainyao H., Chen M.H., Wang W.S. (2000). Antibacterial effect of chloramphenicol, thiamphenicol and florfenicol against aquatic animal bacteria. J. Vet. Med. Sci..

[12] Shin S.J., Sang G.K., Nabin R., Mi L.K., Han S.Y. (2005). Evaluation of the antimicrobial activity of florfenicol against bacteria isolated from bovine and porcine respiratory disease. Vet. Microbiol..

[13] Wisselink H.J., Veldman K.T. (2006). Quantitative susceptibility of Streptococcus suis strains isolated from diseased pigs in seven European countries to antimicrobial agents licenced in veterinary medicine. Vet. Microbiol..

[14] Zhang Q., Tang S.S., Qian M.M., Wei L., Zhou D., Zhang Z.Z., He J.K., Zhang Q.J., Zhu P., Xiao X.L. (2016). Nanoemulsion formulation of florfenicol improves bioavailability in pigs. J. Vet. Pharmacol. Ther..

[15] Liu C., Wang S.J., Zhang Q., Shao Y.X. (2015). Influence of three coccidiostats on the pharmacokinetics of florfenicol in rabbits. Exp. Anim. Tokyo.

[16] Wang G.Y., Tu P., Chen X., Guo Y.G., Jiang S.X. (2013). Effect of three polyether ionophores on pharmacokinetics of florfenicol in male broilers. J. Vet. Pharmacol. Ther..

[17] Shen J., Hu D., Wu X., Coats J.R. (2010). Bioavailability and pharmacokinetics of florfenicol in broiler chickens. J. Vet. Pharmacol. Ther..

[18] Liu N., Guo M., Mo F., Sun Y.Y., Yuan Z., Cao L.L., Jiang S.X. (2012). Involvement of p-glycoprotein and cytochrome p450 3a in the metabolism of florfenicol of rabbits. J. Vet. Pharmacol. Ther..

[19] Wang G.Y., Zheng H.H., Zhang K.Y., Yang F., Kong T., Zhou B., Jiang S.X. (2018). The roles of cytochrome p450 and p-glycoprotein in the pharmacokinetics of florfenicol in chickens. Iran. J. Vet. Res..

[20] Laszlo L., Sarkadi B., Hegedus T. (2016). Jump into a New Fold—A Homology Based Model for the ABCG2/BCRP Multidrug Transporter. PLoS ONE.

[21] Keskitalo J.E., Zolk O., Fromm M.F., Kurkinen K.J., Neuvonen P.J., Niemi M. (2009). Abcg2 polymorphism markedly affects the pharmacokinetics of atorvastatin and rosuvastatin. Clin. Pharmacol. Ther..

[22] Keskitalo J.E., Pasanen M.K., Neuvonen P.J., Niemi M. (2009). Different effects of the abcg2 c.421c>a snp on the pharmacokinetics of fluvastatin, pravastatin and simvastatin. Pharmacogenomics.

[23] Xu Y.J., Wang Y., Lu Y.F., Xu S.F., Wu Q., Liu J. (2017). Age-associated differences in transporter gene expression in kidneys of male rats. Mol. Med. Rep..

[24] Riches Z., Abanda N., Collier A.C. (2015). BCRP protein levels do not differ regionally in adult human livers, but decline in the elderly. Chemico-Biol. Interact..

[25] Konieczna A., Erdösová B., Lichnovská R., Jandl M., Čížková K., Ehrmann J. (2011). Differential expression of abc transporters (mdr1, mrp1, bcrp) in developing human embryos. J. Mol. Histol..

[26] Demeule M., Jodoin J., Beaulieu E., Brossard M., Béliveau R. (1999). Dexamethasone modulation of multidrug transporters in normal tissues. FEBS Lett..

[27] Iqbal M., Baello S., Javam M., Audette M.C., Gibb W., Matthews S.G. (2015). Regulation of multidrug resistance p-glycoprotein in the developing blood-brain barrier: Interplay between glucocorticoids and cytokines. J. Neuroendocrinol..

[28] Zhang Y., Huang J., Liu Y., Guo T., Wang L. (2018). Using the lentiviral vector system to stably express chicken p-gp and bcrp in mdck cells for screening the substrates and studying the interplay of both transporters. Arch. Toxicol..

[29] Shi Z., Parmar S., Peng X.X., Shen T., Robey R.W., Bates S.E., Fu L.W., Shao Y., Chen Y.M., Zang F. (2009). The epidermal growth factor tyrosine kinase inhibitor AG1478 and erlotinib reverse ABCG2-mediated drug resistance. Oncol. Rep..

[30] Ismail M., Elkattan Y.A. (2009). Comparative pharmacokinetics of florfenicol in the chicken, pigeon and quail. Br. Poult. Sci..

[31] Anadón A., Martínez M.A., Martínez M., Ríos A., Caballero V., Ares I., Martínez-Larrañaga M.R. (2008). Plasma and tissue depletion of florfenicol and florfenicol-amine in chickens. J. Agric. Food Chem..

[32] Anonymous Committee for Veterinary Medicinal Products; Florfenicol (Extension to Chicken), Summary Report 3; EMEA/MRL/598/99-FINAL; 1999. http://www.ema.europa.eu/ema/pages/includes/document/open_document.jsp?webContentId=WC500014277.

[33] Sunghwan K., Thiessen P.A., Bolton E.E., Jie C., Gang F., Asta G., Han L.Y., He J., He S.Q. (2016). Pubchem substance and compound databases. Nucleic Acids Res..

[34] Hanwell M.D., Curtis D.E., Lonie D.C., Vandermeersch T., Zurek E., Hutchison G.R. (2012). Avogadro: An advanced semantic chemical editor, visualization, and analysis platform. J. Cheminform..

[35] Bhutto Z.A., He F., Zloh M., Yang J., Huang J., Guo T., Wang L. (2018). Use of quercetin in animal feed: Effects on the p-gp expression and pharmacokinetics of orally administrated enrofloxacin in chicken. Sci. Rep..

[36] Bertoni M., Kiefer F., Biasini M., Bordoli L., Schwede T. (2017). Modeling protein quaternary structure of homo- and hetero-oligomers beyond binary interactions by homology. Sci. Rep..

[37] Biasini M., Bienert S., Waterhouse A., Arnold K., Studer G., Schmidt T., Kiefer F., Gallo Cassarino T., Bertoni M., Bordoli L. (2014). Swiss-model: Modelling protein tertiary and quaternary structure using evolutionary information. Nucleic Acids Res..

[38] Jackson S.M., Manolaridis I., Kowal J., Zechner M., Taylor N.M.I., Bause M., Bauer S., Bartholomaeus R., Bernhardt G., Koenig B. (2018). Structural basis of small-molecule inhibition of human multidrug transporter abcg2. Nat. Struct. Mol. Biol..

[39] Osguthorpe D.J., Sherman W., Hagler A.T. (2012). Exploring Protein Flexibility: Incorporating Structural Ensembles from Crystal Structures and Simulation into Virtual Screening Protocols. J. Phys. Chem. B.

[40] Bowers K.J., Chow E., Xu H., Dror R.O., Eastwood M.P., Gregersen B.A., Klepeis J.L., Kolossvary I., Moraes M.A., Sacerdoti F.D. Scalable Algorithms for Molecular Dynamics Simulations on Commodity Clusters. Proceedings of the ACM/IEEE Conference on Supercomputing.

[41] Trott O., Olson A.J. (2010). AutoDock Vina: Improving the speed and accuracy of docking with a new scoring function, efficient optimization, and multithreading. J. Comput. Chem..

[42] Pedretti A., Villa L., Vistoli G. (2004). VEGA—An open platform to develop chemo-bio-informatics applications, using plug-in architecture and script programming. J. Comput. Aided Mol. Des..

[43] Livak K.J., Schmittgen T.D. (2001). Analysis of relative gene expression data using real-time quantitative PCR and the 2(-Delta Delta C (T)) Method. Methods.

